# Adherence to STOMP principles in CAMHS learning disability services: a clinical audit of prescribing, monitoring, and multidisciplinary practice

**DOI:** 10.1192/j.eurpsy.2026.11149

**Published:** 2026-07-31

**Authors:** B. T. Kece, K. Powell

**Affiliations:** Psychiatry, Herefordshire and Worcestershire Health and Care NHS Trust, Hereford, United Kingdom

## Abstract

**Introduction:**

Psychotropic over-prescription in people with learning disability remains a global concern. STOMP promotes lowest-effective-dose prescribing, psychosocial support, and regular review. We audited two CAMHS LD services (Hereford and Worcester) against STOMP standards.

**Objectives:**

To compare: (1) documentation of indication and reduce/stop plans; (2) specialist initiation; (3) MDT involvement at initiation and review; (4) review frequency; (5) antipsychotic monitoring; (6) behavioural/psych support; and (7) capacity/best-interests documentation.

**Methods:**

Retrospective case-note review using a standard pro-forma. Hereford: 20 young people (8-17y) and Worcester: 12 young people (8-17y) with moderate-severe LD with or without autism on psychotropics were randomly selected. Data was collected on: specialist initiation, reduce/stop plan, MDT input, review interval, behavioural plan, capacity/best-interests and rationale documentation along baseline/ongoing monitoring.

**Results:**

Prescribing was specialist-initiated with rationale documented in both counties (Hereford, 90%; Worcester, 100%). Reduce/stop plans were rare (Hereford, 10%; Worcester, 17%). MDT involvement at initiation was limited in Hereford (30%) but registered more in Worcester (67%). Ongoing MDT reviews were absent in both counties. Antipsychotic use was higher in Hereford (70%) than Worcester (50%). Monitoring was complete for most in Hereford (79%) and Worcester (83%). Capacity/best-interest documentation in Worcester was fully consistent (100%) but lower in Hereford (60%). Review intervals aligned with guidance.

**Table 1.** Comparative findings Table 1. Long description.

Metric	Hereford (N=20)	Worcester (N=12)
Rationale documented	18/20 (90%)	12/12 (100%)
Reduce/stop plan	2/20 (10%)	2/12 (17%)
MDT at initiation	6/20 (30%)	8/12 (67%)
Ongoing MDT review	0/20 (0%)	0/12 (0%)
Review <=6 months	20/20 (100%)	12/12 (100%)
Antipsychotic use	14/20 (70%)	6/12 (50%)
Monitoring complete	11/14 (79%)	5/6 (83%)
Behavioural/psych plan	6/20 (60%)	5/12 (41%)
Capacity/best-interests	12/20 (60%)	12/12 (100%)

**Conclusions:**

Implementing the same audit in two CAMHS LD services revealed shared strengths (specialist initiation, clear rationale, timely reviews) and persistent gaps (limited de-prescribing plans, variable behavioural support, incomplete monitoring/documentation for a minority). Differences likely reflect local structures: Worcester showed stronger initiation stage with MDT discussions and capacity/best-interests recording, whereas Hereford showed clearer antipsychotic monitoring completion. Quality-improvement actions include embedding a mandatory “medication review” field in MDT pro forma, scheduled MDT follow-ups, standardised monitoring systems within the Trust, and strengthening pathways to psychological and behavioural support. These recommendations align with NHS England STOMP guidance.

**Disclosure of Interest:**

None Declared

## Long descriptions

### Table 1. Long description

The table consists of three columns: Metric, Hereford (N equals 20), and Worcester (N equals 12).

* Rationale documented: Hereford 18/20 (90 percent); Worcester 12/12 (100 percent).

* Reduce/stop plan: Hereford 2/20 (10 percent); Worcester 2/12 (17 percent).

* M D T at initiation: Hereford 6/20 (30 percent); Worcester 8/12 (67 percent).

* Ongoing M D T review: Hereford 0/20 (0 percent); Worcester 0/12 (0 percent).

* Review less than or equal to 6 months: Hereford 20/20 (100 percent); Worcester 12/12 (100 percent).

* Antipsychotic use: Hereford 14/20 (70 percent); Worcester 6/12 (50 percent).

* Monitoring complete: Hereford 11/14 (79 percent); Worcester 5/6 (83 percent).

* Behavioural/psych plan: Hereford 6/20 (60 percent); Worcester 5/12 (41 percent).

* Capacity/best-interests: Hereford 12/20 (60 percent); Worcester 12/12 (100 percent).

Navigate back to Table 1..

